# pH-Dependent entry of chikungunya virus fusion into mosquito cells

**DOI:** 10.1186/s12985-014-0215-y

**Published:** 2014-12-05

**Authors:** John T Nuckols, Alexander J McAuley, Yan-Jang S Huang, Kate M Horne, Stephen Higgs, Robert A Davey, Dana L Vanlandingham

**Affiliations:** Medical Countermeasure Systems, Joint Vaccine Acquisition Program, Fort Detrick, MD 21702 USA; Department of Microbiology and Immunology, University of Texas Medical Branch, Galveston, Texas 77555 USA; Biosecurity Research Institute, Kansas State University, Manhattan, Kansas 66506 USA; Department of Diagnostic Medicine/Pathobiology, Kansas State University, Manhattan, KS 66506 USA; Texas Biomedical Research Institute, San Antonio, TX 78245 USA

**Keywords:** Chikungunya virus, Lysosomotropic compounds, Virus entry

## Abstract

**Background:**

Millions of human infections caused by arthropod-borne pathogens are initiated by the feeding of an infected mosquito on a vertebrate. However, interactions between the viruses and the mosquito vector, which facilitates successful infection and transmission of virus to a subsequent vertebrate host, are still not fully understood.

**Finding:**

Here we describe early chikungunya virus (CHIKV) infectious events in cells derived from one of the most important CHIKV vectors, *Aedes albopictus*. We demonstrated that CHIKV infection of mosquito cells depended on acidification of the endosome as indicated by significant inhibition following prophylactic treatment with the lysosomotropic drugs chloroquine, ammonium chloride, and monensin, which is consistent with observations in mammalian cells. While all three agents inhibited CHIKV infection in C6/36 cells, ammonium chloride was less toxic to cells than the other agents.

**Conclusion:**

The observation of similar mechanisms for inhibition of CHIKV infection in mosquito and mammalian cell lines suggests that conserved entry pathways are utilized by CHIKV for vertebrate and invertebrate cell types.

The mechanism of entry of chikungunya virus (CHIKV) into mosquito cells has not been well studied although the pH-dependent nature of CHIKV entry into the cytoplasm from the endosome has been characterized in mammalian cells using chloroquine, ammonium chloride (NH_4_Cl), or monensin in Vero and HEK293T cell lines [1,2]. Examination of the effect of these drugs on CHIKV infection of C6/36 cells provided a unique opportunity to evaluate this model in a cell line derived from the CHIKV mosquito vector, *Aedes albopictus* [3]. CHIKV is an alphavirus that was transmitted in nature primarily by *Aedes aegypti* up until approximately 2005 when a point mutation of alanine to valine at codon 226 in the E1 protein enhanced infection of and transmission by *Ae. albopictus* mosquitoes [4]. Typically, CHIKV re-emerges within endemic regions approximately every 7–20 years before retreating to an unknown sylvatic maintenance host [5,6]. The recent outbreaks caused severe illness in the Indian Ocean islands in 2005, most notably Reunion where 260 deaths were attributed to CHIKV infection and India where at least 1.4 million persons have acquired the infection, and the continued dispersion of the virus by travelers to and from these regions have brought this virus to the forefront of potentially severe pathogenic agents [4,7]. Since 2013, an outbreak on Caribbean Islands has resulted in an estimated 790,000 cases, with over 1500 imported cases in the US, and transmission cycles occurring in many South and Central American countries [8].

Alphaviruses demonstrate significant inter- and intra-clade differences regarding the regulation of their specific mechanisms of cellular entry into mammalian and mosquito cells [9-11]. Thus, it is necessary to determine the mechanistic processes used by specific viruses to understand the etiology of infections. Chloroquine and NH_4_Cl affect endocytic pH levels by accumulating in protonated forms within the acidic compartments of cells, effectively binding H^+^ ions, raising the pH of the endosomes and inhibiting pH-dependent viral membrane fusion [12,13]. Monensin is a cationic ionophore with the capacity to neutralize the acidic environment of the endosomes through the endosome membrane [14]. Importantly, these agents have been shown to impede virus particle fusion to the endosome by inhibiting the acidification that dissociates E1/E2 dimers and exposes the E1 fusion peptide [15,16]. While the alphaviruses share the fusion mechanism mediated by the class II fusion proteins, the kinetics of alphavirus fusion during lysosomotropic pre-treatment or treatment is not consistent. Both chloroquine and NH_4_Cl have proven effective at inhibiting Sindbis virus (SINV) infection of BHK-21 cells [9], but in mosquito cells, only NH_4_Cl inhibits SINV infection while chloroquine actually increases infection [17]. Our experiments with CHIKV investigated the prophylactic treatment of C6/36 cells with chloroquine, NH_4_Cl, or monensin, and whether or not this treatment inhibited the replication of a GFP-expressing infectious clone of CHIKVLR2006 OPY1 strain whose infectivity and replication *in vitro* and *in vivo* is similar to wild-type virus [18].

CHIKV entry experiments were performed *in vitro* with C6/36 cells in Leibovitz (L-15) medium incubated at 28°C overnight to approximately 80% confluence. Chloroquine, NH_4_Cl, or monensin (initial dilutions of 50 μM, 20 mM, and 20 μM, respectively with eight sequential 2-fold dilutions) were applied to C6/36 cells and incubated for 1 h at 28°C as previously described by Colpitts *et al.* [13]. The mock group for the chemical treatment received a comparable volume of L-15 medium and was grown under the same conditions and in parallel as the untreated cells. Administration of lysosomotropic agents concurrent with, or shortly after, CHIKV infection were not considered for these experiments since it has previously been demonstrated that any form of therapeutic treatment other than prophylactic is less than optimal [2,19]. Following chemical pre-treatment, CHIKVLR2006-OPY1GFP (10^6.95^ TCID_50_/mL) was added to the C6/36 monolayer for a multiplicity of infection (MOI) = 0.5 [18]. This MOI was based upon previous data for SINV which indicated the inhibition of viral entry by lysosomotropic agents could be diminished at MOI of 10 or greater [17,20]. Plates were incubated with CHIKV LR2006-OPY1GFP virus for 1 h at 28°C, cell culture media was then replaced with fresh media. Monolayers were incubated for eight-hours at 28°C, media was removed, and cells were fixed overnight in 10% formalin. Uninfected, treated and non-treated plates were washed three times with Dulbecco’s phosphate buffered saline (DPBS) followed by application of Alamar Blue (Invitrogen, Carlsbad, CA) to assess cell survival and to monitor for deleterious drug treatment effects on the C6/36 cells (data not shown). CHIKV infected plates were washed three times with DPBS followed by the application of 4′, 6-diamidino-2-phenylindole (DAPI). Prophylactic treatment of C6/36 cells with chloroquine and NH_4_Cl was well tolerated at all concentrations tested. Monensin pretreatment was also tolerated well by the C6/36 cells with the exception of the two highest concentrations tested (10 and 20 μM). Plates were imaged using the Nikon Eclipse T*i* – Perfect Focus system inverted microscope and analyzed with Cell Profiler software to quantify degree of infection based on the GFP expression. Data were analyzed using a one-way ANOVA with a Tukey post-hoc test.

In these experiments, C6/36 cells were pre-treated for 1 h with a range of 2-fold concentrations of chloroquine (0.781-100 μM), NH_4_Cl (0.156-20 mM), or monensin (0.156-20 μM) followed by a one-hour viral absorption period and an eight-hour long incubation period to allow CHIKV replication and GFP expression. In the absence of lysosomotropic agents, 38.6% of the total cell population in the mock group was positive for virus-induced GFP expression. An average survival rate of 86.3% was recorded for untreated, CHIKV-infected cells in comparison to C6/36 control cells seeded at the same density and incubated without the chemical treatments or the infection of CHIKV. A significant decrease in infection (*p* < 0.005) was seen with concentrations >25 μM for chloroquine, >1.25 mM for NH_4_Cl and >1.25 μM for monensin (Figure 1). The effective dose_50_ (ED_50_), i.e. the amount of compound that resulted in a 50% reduction of CHIKV infection of treated C6/36 cells, was estimated to be 26.5 μM, 0.85 mM and 0.95 μM for chloroquine, NH_4_Cl and monensin, respectively (Figure 1). The treatment of lysosomotropic compounds did not result in the significant loss of cell viability except for the treatment of monensin at high concentrations. The adverse effect of monensin treatment was observed at the concentrations greater than 5 μM, which led to significantly higher mortality (*p* < 0.005) than the control group. The percentage of survival in C6/36 cells treated with chloroquine and NH_4_Cl was equivalent to untreated controls.

**Figure 1:**
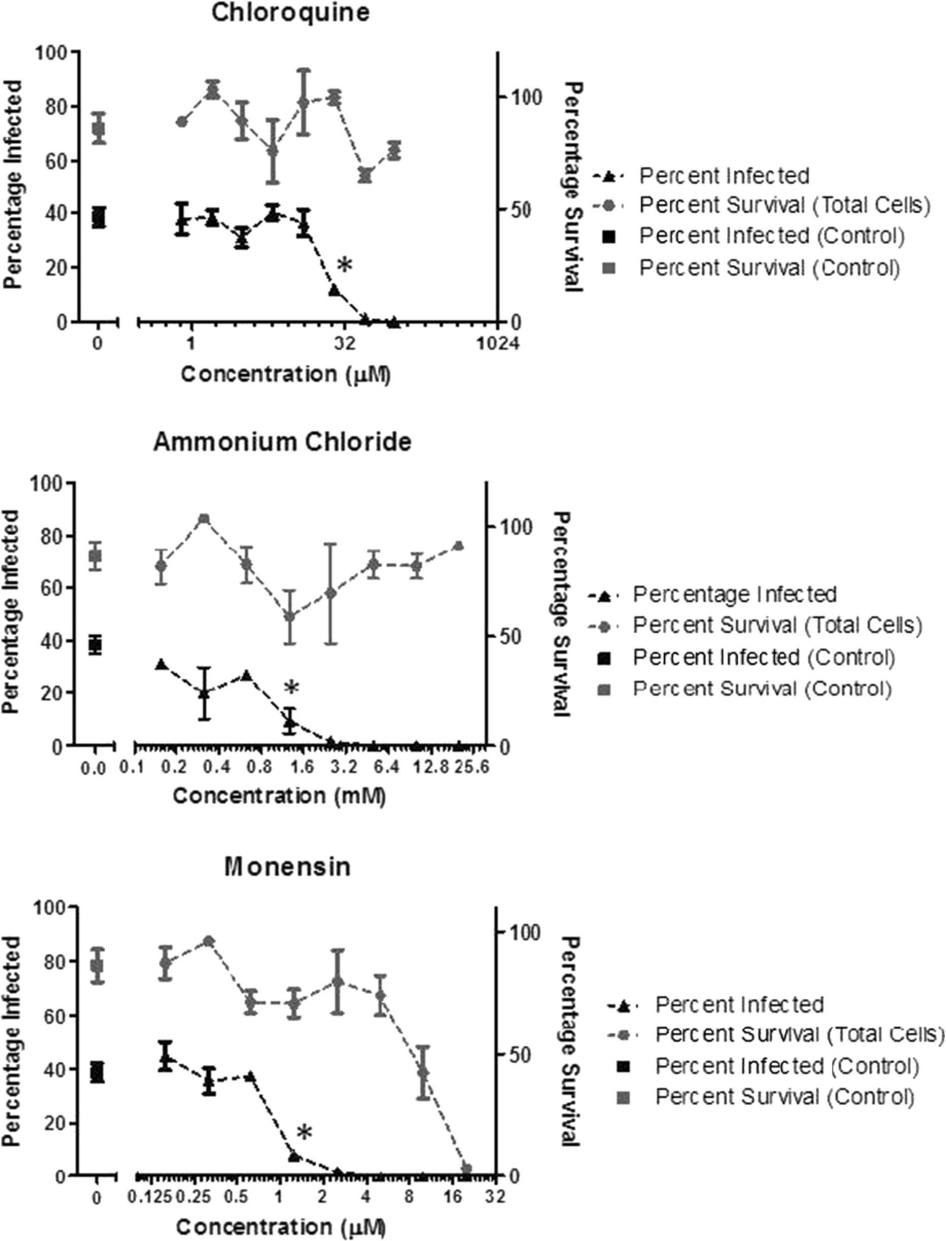
**C6/36 mosquito cell survival and CHIKV infection rates in the presence of lysosomotropic agents.** C6/36 cells were treated with serially diluted lysosomotropic agents prior to the infection of CHIKVLR2006-OPY1 GFP. The concentrations of each dilution are shown on the x-axis. After eight hours of propagation, virus entry was quantified by the percentage of GFP-positive cells (black triangle, left *y*-axis). The lowest concentration of each lysosomotropic agent that leads to statistically significant reduction of CHIKV entry is highlighted with asterisks. The cytotoxicity was monitored by Alamar blue staining and quantified as percentage of survival (grey circle, right *y*-axis). Grey squares represent percentages of survival for C6/36 cells that were not subjected to the treatment of lysosomotropic agents and the infection of CHIKV. Black squares represent the percentage of infected C6/36 cells without the treatment of lysosomotropic agents.

The results of this study suggest CHIKV viral entry in mosquito cells is highly pH-dependent and the selective blockade of acidification during endocytosis can be an effective strategy to block viral entry. This study also suggests that the quantity of chloroquine necessary to inhibit CHIKV entry in C6/36 cells was similar to the quantity required for CHIKV entry inhibition in Vero cells. The use of chloroquine and NH_4_Cl present optimal prophylactic treatment models because of relatively lower concentrations required to suppress CHIKV infection, the absence of cytotoxic events, and the similar inhibition of CHIKV infection in Vero cells [2].

Previous studies of SINV entry in *Ae. albopictus* cells did not record significant entry inhibition mediated by chloroquine treatment at 100 mM and 5 mM [17,20]. However, we demonstrate here that CHIKV entry can be inhibited by chloroquine at the lower concentration of 26.5 μM. The effect of both chloroquine and NH_4_Cl on CHIKV entry into C6/36 is similar to the inhibition of the Semliki Forest virus (SFV) and Venezuelan equine encephalitis virus (VEEV) in C710 cells described by Colpitts *et al.* [13]. Although monensin also exhibited inhibitory effects against CHIKV infection, the ED_50_ was observed at concentrations close to those at which increased C6/36 mortality was observed.

In conclusion, results presented here combined with previously published studies indicate suppression of endosomal acidification can be an effective strategy for inhibiting cellular infection of at least three alphaviruses, CHIKV, SFV and VEEV based on the known mechanisms of two primary amine compounds, chloroquine and NH_4_CI. Disruption of the sodium/potassium gradient can potentially cause adverse effects because of the cytotoxicity observed in the treatment of monensin. In addition to incorporating selective blockade of the endocytosis pathway for the design of antiviral compounds, genetic manipulation of vector insects targeting the genes associated with the endocytosis pathways may also serve as a strategy for development of refractory variants of arthropod vectors.
